# Supplementary material for: Identification of Circulating Serum miRNAs as Novel Biomarkers in Pancreatic Cancer Using a Penalized Algorithm
Source: Int J Mol Sci. 2021 Jan 20;22(3):1007. doi: 10.3390/ijms22031007 (PMC7863930; doi:10.3390/ijms22031007)
Supplement: Supplementary file 1 [file ijms-22-01007-s001.pdf]

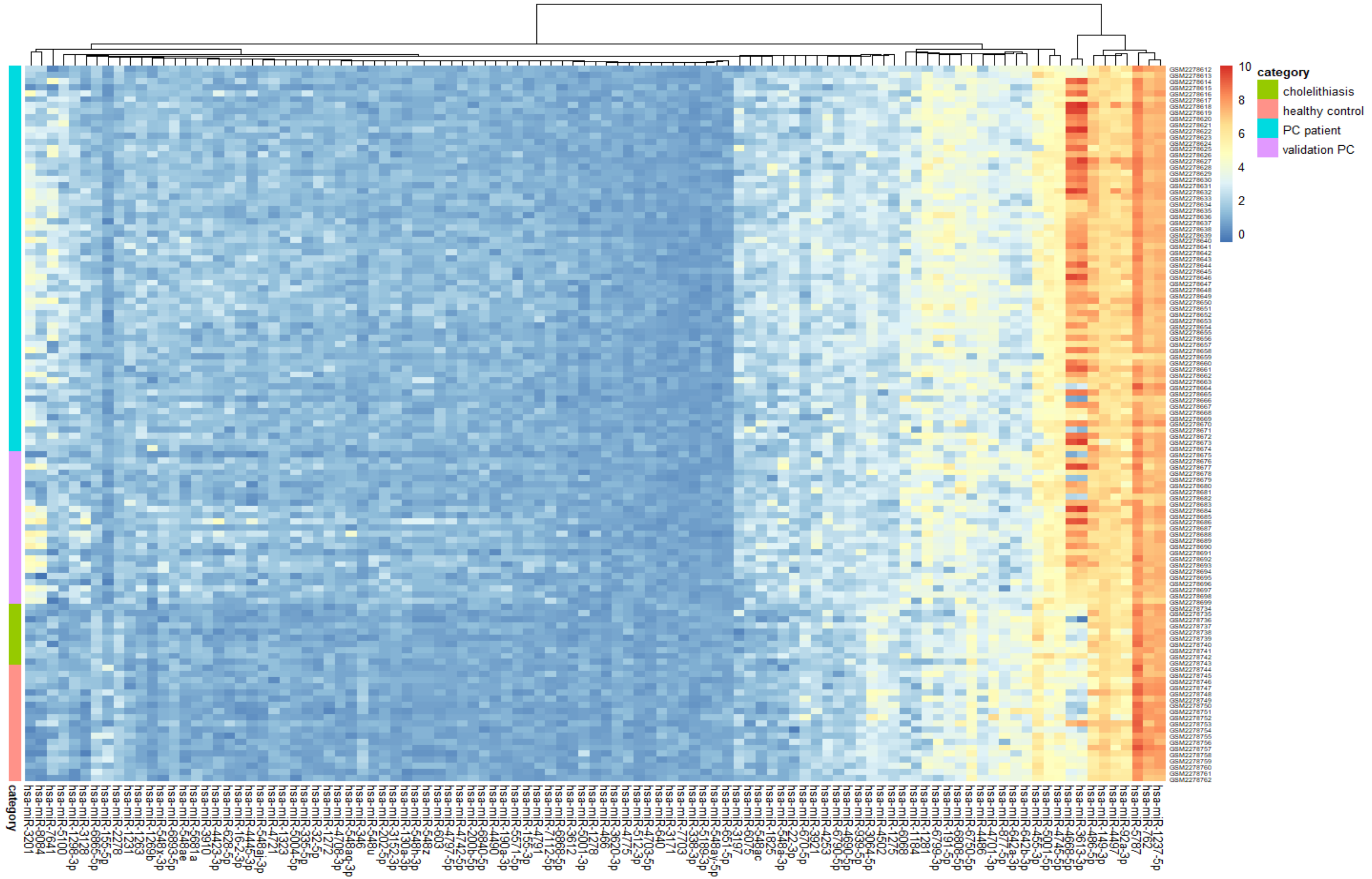

Figure S1. The heatmap of 103 miRNAs for distinguishing PC and non-cancer samples.

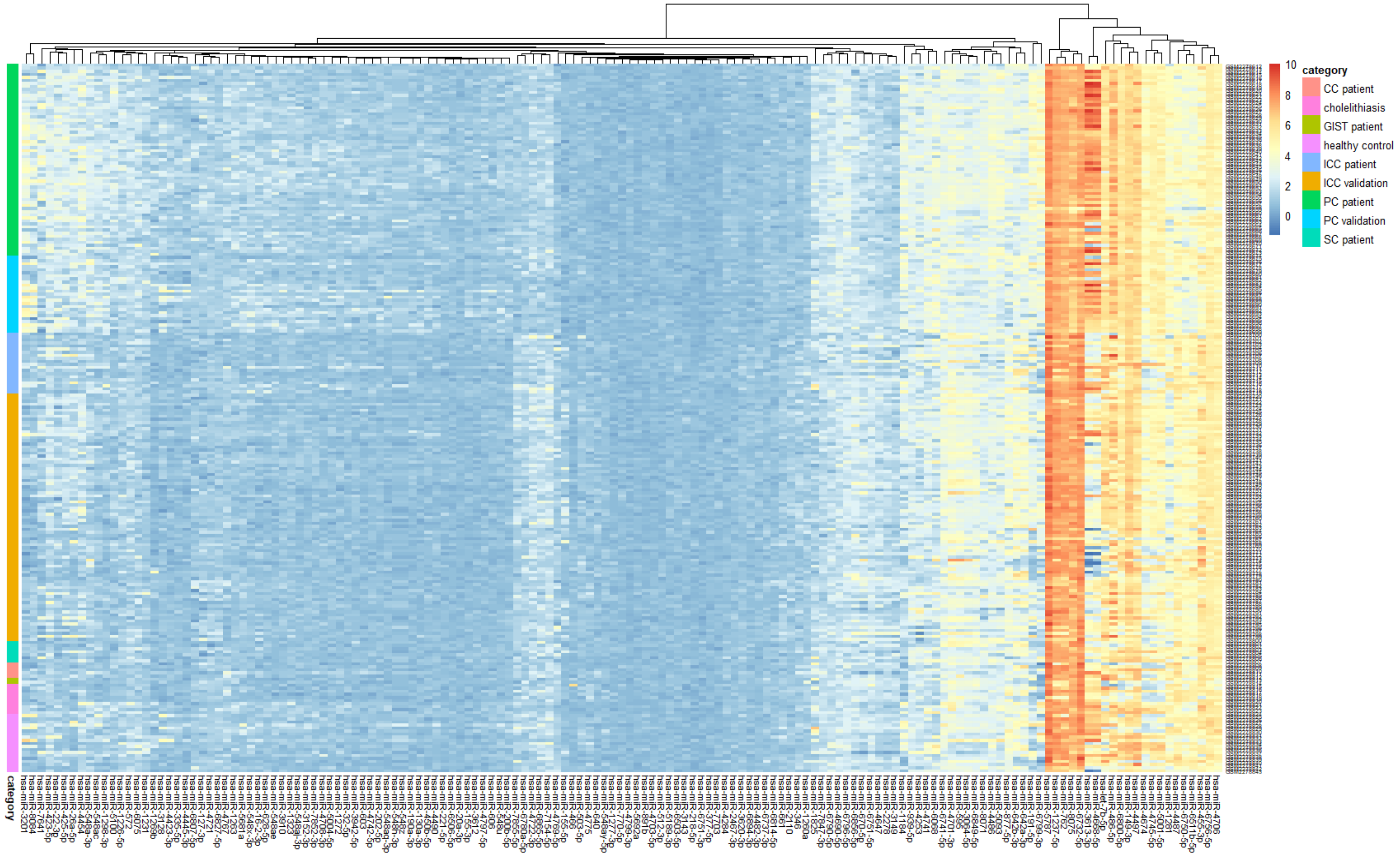

Figure S2. The heatmap of 149 miRNAs for distinguishing PC and non-PC samples.

Table S1. The differentially expressed 103 miRNAs for distinguishing PC and non-cancer samples.

miRNA	Z-value	p-val	FDR	AUC
hsa-miR-4668-5p*	-5.060	4.19E-07	0.000584	0.910783
hsa-miR-3613-3p*	-5.045	4.53E-07	0.000584	0.903667
hsa-miR-548ac*	-4.610	4.02E-06	0.002278	0.90312
hsa-miR-1298-3p*	-4.604	4.15E-06	0.002278	0.906951
hsa-miR-16-2-3p*	-4.580	4.66E-06	0.002278	0.86809
hsa-miR-4708-3p*	-4.553	5.30E-06	0.002278	0.873563
hsa-miR-548a-3p*	-4.467	7.93E-06	0.002919	0.835249
hsa-miR-3201*	-4.418	9.97E-06	0.003155	0.925014
hsa-miR-8084*	-4.397	1.10E-05	0.003155	0.86809
hsa-miR-548aq-3p*	-4.321	1.55E-05	0.003244	0.834702
hsa-miR-628-5p*	-4.264	2.01E-05	0.003244	0.847291
hsa-miR-455-3p*	4.262	2.03E-05	0.003244	0.836891
hsa-miR-548h-3p*	-4.248	2.16E-05	0.003244	0.834154
hsa-miR-548z*	-4.248	2.16E-05	0.003244	0.834154
hsa-miR-32-5p*	-4.243	2.21E-05	0.003244	0.831418
hsa-miR-22-3p*	-4.240	2.24E-05	0.003244	0.819923
hsa-miR-1269b*	-4.227	2.37E-05	0.003244	0.904762
hsa-miR-346*	4.225	2.39E-05	0.003244	0.793651
hsa-miR-3910*	-4.225	2.39E-05	0.003244	0.843459
hsa-miR-4797-5p*	-4.209	2.57E-05	0.003244	0.853859
hsa-miR-1263*	-4.202	2.64E-05	0.003244	0.837438
hsa-miR-548ae*	-4.133	3.58E-05	0.004194	0.840175
hsa-miR-642a-3p*	4.099	4.15E-05	0.004475	0.800766
hsa-miR-1184*	-4.096	4.20E-05	0.004475	0.808976
hsa-miR-1825*	-4.089	4.34E-05	0.004475	0.81445
hsa-miR-762*	4.042	5.31E-05	0.005055	0.760263
hsa-miR-5100*	-4.038	5.39E-05	0.005055	0.792009
hsa-miR-335-5p*	-4.030	5.58E-05	0.005055	0.834702
hsa-miR-548aj-3p*	-4.025	5.69E-05	0.005055	0.789272
hsa-miR-1272*	-3.993	6.52E-05	0.005605	0.793103
hsa-miR-486-5p*	-3.976	7.01E-05	0.005753	0.781609
hsa-miR-642b-3p*	3.972	7.14E-05	0.005753	0.789819
hsa-miR-6865-5p*	3.919	8.90E-05	0.006797	0.776136
hsa-miR-4423-3p*	-3.912	9.15E-05	0.006797	0.80405
hsa-miR-3128*	-3.910	9.23E-05	0.006797	0.80624
hsa-miR-548x-3p*	-3.886	0.000102	0.007318	0.812808
hsa-miR-1281*	-3.879	0.000105	0.007318	0.798577
hsa-miR-603*	-3.872	0.000108	0.007344	0.793651

hsa-miR-4486*	-3.859	0.000114	0.007516	0.788725
hsa-miR-548u*	-3.844	0.000121	0.007652	0.77723
hsa-miR-640*	3.842	0.000122	0.007652	0.746579
hsa-miR-4742-5p*	-3.828	0.000129	0.007943	0.791461
hsa-miR-3152-3p*	-3.810	0.000139	0.008337	0.782157
hsa-miR-1323*	-3.793	0.000149	0.008618	0.772852
hsa-miR-5787*	3.791	0.00015	0.008618	0.755884
hsa-miR-6068*	-3.758	0.000171	0.009597	0.77723
hsa-miR-6840-5p	-3.751	0.000176	0.009669	0.786535
hsa-miR-6750-5p*	3.729	0.000192	0.010302	0.785441
hsa-miR-5681a*	-3.713	0.000205	0.010634	0.765736
hsa-miR-548ay-5p*	3.712	0.000206	0.010634	0.773946
hsa-miR-6075*	-3.702	0.000214	0.010841	0.762452
hsa-miR-4502	3.679	0.000234	0.011623	0.756979
hsa-miR-3064-5p*	3.614	0.000301	0.014624	0.768473
hsa-miR-5001-5p*	-3.604	0.000313	0.014935	0.775588
hsa-miR-155-3p*	-3.593	0.000327	0.01525	0.770115
hsa-miR-155-5p*	3.590	0.000331	0.01525	0.843459
hsa-miR-1237-5p*	3.569	0.000358	0.016206	0.733443
hsa-miR-7641*	-3.547	0.00039	0.017356	0.759715
hsa-miR-5004-5p*	-3.542	0.000397	0.017365	0.764641
hsa-miR-4690-5p*	-3.496	0.000472	0.020262	0.747674
hsa-miR-4497*	-3.492	0.00048	0.020293	0.74439
hsa-miR-4745-5p*	-3.458	0.000545	0.022645	0.721949
hsa-miR-4703-5p*	-3.429	0.000606	0.024817	0.725233
hsa-miR-5189-3p*	3.414	0.00064	0.025762	0.722496
hsa-miR-512-3p*	-3.392	0.000693	0.02687	0.737274
hsa-miR-939-5p*	-3.391	0.000696	0.02687	0.720854
hsa-miR-4445-3p*	-3.390	0.000698	0.02687	0.759715
hsa-miR-2278*	3.364	0.000768	0.029108	0.718664
hsa-miR-1231*	-3.352	0.000802	0.02994	0.728517
hsa-miR-3612*	-3.348	0.000813	0.02994	0.735085
hsa-miR-6790-5p*	-3.338	0.000844	0.030633	0.726327
hsa-miR-877-5p*	3.330	0.00087	0.031163	0.739464
hsa-miR-6808-5p	-3.319	0.000904	0.03193	0.747674
hsa-miR-3620-3p*	3.315	0.000917	0.031947	0.723043
hsa-miR-6893-5p	-3.311	0.000931	0.032001	0.721401
hsa-miR-1275	3.299	0.000971	0.032941	0.689655
hsa-miR-466*	-3.293	0.00099	0.033141	0.738369
hsa-miR-190a-3p*	-3.267	0.001087	0.035934	0.723591
hsa-miR-200b-5p*	-3.262	0.001107	0.036111	0.727969

hsa-miR-4721*	-3.253	0.001143	0.036836	0.755884
hsa-miR-4490*	-3.242	0.001187	0.037766	0.7526
hsa-miR-3197	-3.238	0.001203	0.037824	0.721401
hsa-miR-191-5p*	-3.222	0.001275	0.039517	0.76902
hsa-miR-3171	3.218	0.001293	0.039517	0.719212
hsa-miR-92a-3p	-3.213	0.001315	0.039517	0.712096
hsa-miR-651-5p	3.212	0.001318	0.039517	0.732895
hsa-miR-5571-5p	-3.206	0.001344	0.039694	0.724685
hsa-miR-6799-3p*	-3.201	0.001369	0.039694	0.70717
hsa-miR-202-5p*	-3.201	0.00137	0.039694	0.730159
hsa-miR-149-3p*	3.192	0.001413	0.040478	0.718664
hsa-miR-670-5p*	3.182	0.001462	0.041422	0.71538
hsa-miR-5001-3p	3.178	0.001483	0.041543	0.732895
hsa-miR-130a-3p*	-3.171	0.001521	0.042071	0.711549
hsa-miR-7703*	3.168	0.001534	0.042071	0.68254
hsa-miR-3921	-3.162	0.001565	0.042228	0.73399
hsa-miR-4791	-3.161	0.001573	0.042228	0.723043
hsa-miR-338-3p	3.156	0.001601	0.042553	0.702791
hsa-miR-4253*	-3.147	0.00165	0.043398	0.713738
hsa-miR-6868-5p	-3.125	0.00178	0.046361	0.738369
hsa-miR-7112-5p	-3.120	0.001811	0.046697	0.747674
hsa-miR-4701-3p*	3.102	0.001922	0.048675	0.701149
hsa-miR-1278	-3.101	0.001926	0.048675	0.720307
hsa-miR-4775*	3.098	0.001948	0.048758	0.70717

*the markers that were also differentially expressed between PC and non-PC samples; other markers were only differentially expressed between PC and non-cancer samples.

Table S2. The differentially expressed 149 miRNAs for distinguishing PC and non-PC samples.

miRNA	Z-value	p-val	FDR	AUC
hsa-miR-3201*	-5.827	5.63E-09	1.45E-05	0.867977
hsa-miR-3613-3p*	-5.707	1.15E-08	1.49E-05	0.825901
hsa-miR-4668-5p*	-5.585	2.34E-08	2.01E-05	0.823381
hsa-miR-548ac*	-5.452	4.98E-08	3.21E-05	0.847317
hsa-miR-1269b*	-5.261	1.43E-07	7.35E-05	0.85941
hsa-miR-8084*	-5.152	2.58E-07	0.000111	0.813555
hsa-miR-548a-3p*	-5.105	3.30E-07	0.000122	0.794911
hsa-miR-16-2-3p*	-5.032	4.85E-07	0.00015	0.801209
hsa-miR-1263*	-5.008	5.51E-07	0.00015	0.789872
hsa-miR-22-3p*	-4.994	5.91E-07	0.00015	0.787352
hsa-miR-5100*	-4.979	6.39E-07	0.00015	0.776266
hsa-miR-4708-3p*	-4.928	8.30E-07	0.000178	0.834215
hsa-miR-6865-5p*	4.864	1.15E-06	0.000198	0.77425
hsa-miR-1298-3p*	-4.864	1.15E-06	0.000198	0.797682
hsa-miR-642b-3p*	4.864	1.15E-06	0.000198	0.78962
hsa-miR-642a-3p*	4.786	1.70E-06	0.000274	0.78584
hsa-miR-3910*	-4.745	2.09E-06	0.000316	0.77551
hsa-miR-4486*	-4.692	2.70E-06	0.000387	0.774502
hsa-miR-548aq-3p*	-4.661	3.15E-06	0.000387	0.773998
hsa-miR-548ay-5p*	4.657	3.21E-06	0.000387	0.768204
hsa-miR-155-5p*	4.647	3.37E-06	0.000387	0.817334
hsa-miR-335-5p*	-4.646	3.38E-06	0.000387	0.78962
hsa-miR-3128*	-4.642	3.45E-06	0.000387	0.772235
hsa-miR-3064-5p*	4.589	4.45E-06	0.000478	0.778786
hsa-miR-548ae*	-4.580	4.64E-06	0.000478	0.786596
hsa-miR-548u*	-4.537	5.70E-06	0.000565	0.750063
hsa-miR-6750-5p*	4.528	5.96E-06	0.000569	0.772235
hsa-miR-346*	4.516	6.31E-06	0.000581	0.737969
hsa-miR-4797-5p*	-4.475	7.63E-06	0.000678	0.769967
hsa-miR-32-5p*	-4.379	1.19E-05	0.00102	0.758125
hsa-miR-1323*	-4.336	1.45E-05	0.001206	0.741749
hsa-miR-4742-5p*	-4.291	1.78E-05	0.001434	0.755102
hsa-miR-877-5p*	4.280	1.87E-05	0.001453	0.732678
hsa-miR-628-5p*	-4.274	1.92E-05	0.001453	0.771227
hsa-miR-127-3p	-4.241	2.23E-05	0.001646	0.743512
hsa-miR-3152-3p*	-4.209	2.57E-05	0.001841	0.737717
hsa-miR-4423-3p*	-4.191	2.78E-05	0.00191	0.730411
hsa-miR-4690-5p*	-4.188	2.82E-05	0.00191	0.737969
hsa-miR-548aj-3p*	-4.140	3.47E-05	0.002223	0.72386

hsa-miR-640*	4.138	3.50E-05	0.002223	0.721088
hsa-miR-2278*	4.136	3.54E-05	0.002223	0.727891
hsa-miR-6075*	-4.105	4.04E-05	0.00248	0.731922
hsa-miR-4701-3p*	4.092	4.27E-05	0.00256	0.721844
hsa-miR-1272*	-4.057	4.97E-05	0.002912	0.755102
hsa-miR-455-3p*	4.050	5.13E-05	0.002938	0.732174
hsa-miR-5787*	4.041	5.33E-05	0.002988	0.719829
hsa-miR-200b-5p*	-4.033	5.50E-05	0.003017	0.719073
hsa-miR-7641*	-3.992	6.54E-05	0.003464	0.714286
hsa-miR-6511b-5p	3.991	6.58E-05	0.003464	0.730915
hsa-miR-4741	-3.982	6.84E-05	0.003526	0.703704
hsa-miR-155-3p*	-3.974	7.08E-05	0.003531	0.716049
hsa-miR-6068*	-3.972	7.12E-05	0.003531	0.720585
hsa-miR-4647	3.950	7.80E-05	0.003793	0.734694
hsa-miR-191-5p*	-3.946	7.96E-05	0.003801	0.74704
hsa-miR-548h-3p*	-3.929	8.54E-05	0.003933	0.753842
hsa-miR-548z*	-3.929	8.54E-05	0.003933	0.753842
hsa-miR-939-5p*	-3.919	8.90E-05	0.004027	0.707735
hsa-miR-4284	-3.909	9.28E-05	0.004035	0.710254
hsa-miR-4745-5p*	-3.908	9.31E-05	0.004035	0.707735
hsa-miR-8075	3.906	9.39E-05	0.004035	0.720081
hsa-miR-1281*	-3.865	0.000111	0.004701	0.73293
hsa-miR-5681a*	-3.832	0.000127	0.005283	0.705467
hsa-miR-6894-3p	3.823	0.000132	0.005388	0.699169
hsa-miR-5189-3p*	3.803	0.000143	0.005744	0.702192
hsa-miR-548x-3p*	-3.773	0.000161	0.006376	0.739985
hsa-miR-486-5p*	-3.761	0.000169	0.006517	0.706727
hsa-miR-7154-5p	3.761	0.000169	0.006517	0.707735
hsa-miR-6799-3p*	-3.741	0.000183	0.00695	0.698665
hsa-miR-450b-5p	-3.728	0.000193	0.007198	0.697153
hsa-miR-4775*	3.696	0.000219	0.008078	0.703956
hsa-miR-661	3.690	0.000224	0.008147	0.697657
hsa-miR-1184*	-3.666	0.000246	0.008699	0.709247
hsa-miR-7855-5p	3.666	0.000246	0.008699	0.698665
hsa-miR-6506-5p	-3.657	0.000255	0.008881	0.690854
hsa-miR-221-5p	-3.653	0.000259	0.008891	0.699924
hsa-miR-6751-5p	3.640	0.000273	0.009275	0.689594
hsa-miR-4721*	-3.633	0.00028	0.009275	0.715294
hsa-miR-3620-3p*	3.632	0.000281	0.009275	0.697153
hsa-miR-3942-5p	-3.599	0.000319	0.01042	0.693878
hsa-miR-202-5p*	-3.595	0.000325	0.010459	0.704712

hsa-miR-7852-3p	-3.585	0.000337	0.010717	0.683799
hsa-miR-1237-5p*	3.566	0.000363	0.011406	0.705971
hsa-miR-26a-5p	-3.554	0.000379	0.011782	0.696649
hsa-miR-4445-3p*	-3.542	0.000397	0.012171	0.706475
hsa-miR-503-5p	3.538	0.000403	0.012222	0.713278
hsa-miR-670-5p*	3.535	0.000408	0.012222	0.678256
hsa-miR-5004-5p*	-3.509	0.00045	0.013204	0.679768
hsa-miR-6800-5p	-3.508	0.000451	0.013204	0.68254
hsa-miR-1825*	-3.469	0.000523	0.015107	0.756866
hsa-miR-595	3.464	0.000533	0.015107	0.677501
hsa-miR-6780a-5p	3.464	0.000533	0.015107	0.683547
hsa-miR-149-3p*	3.428	0.000609	0.016975	0.692618
hsa-miR-425-5p	-3.426	0.000612	0.016975	0.678508
hsa-miR-2467-3p	-3.400	0.000675	0.018521	0.672965
hsa-miR-6737-3p	-3.388	0.000705	0.019122	0.684303
hsa-miR-4767	-3.373	0.000744	0.019716	0.672714
hsa-miR-4497*	-3.373	0.000744	0.019716	0.668934
hsa-miR-6849-5p	3.371	0.000749	0.019716	0.683799
hsa-miR-6732-5p	3.338	0.000845	0.021902	0.669186
hsa-miR-4454	-3.336	0.00085	0.021902	0.683044
hsa-let-7b-5p	3.319	0.000902	0.02303	0.674729
hsa-miR-4703-5p*	-3.301	0.000964	0.024212	0.664147
hsa-miR-4487	3.300	0.000967	0.024212	0.690854
hsa-miR-3149	3.295	0.000983	0.024376	0.681028
hsa-miR-762*	3.291	0.000998	0.024502	0.69035
hsa-miR-1277-3p	-3.287	0.001011	0.024577	0.678005
hsa-miR-6856-5p	3.283	0.001027	0.024744	0.704963
hsa-miR-1226-5p	-3.269	0.001081	0.025806	0.68002
hsa-miR-4490*	-3.258	0.001123	0.026571	0.669186
hsa-miR-5692a	3.244	0.001177	0.027429	0.679516
hsa-miR-512-3p*	-3.241	0.001191	0.027429	0.671706
hsa-miR-218-5p	3.241	0.001192	0.027429	0.67095
hsa-miR-1260a	3.233	0.001224	0.027452	0.657092
hsa-miR-4799-3p	-3.233	0.001226	0.027452	0.676745
hsa-miR-603*	-3.231	0.001234	0.027452	0.688335
hsa-miR-7847-3p	-3.231	0.001235	0.027452	0.670446
hsa-miR-1231*	-3.221	0.001278	0.028163	0.652053
hsa-miR-6756-5p	3.191	0.001419	0.031	0.686571
hsa-miR-4674	-3.185	0.001446	0.031132	0.662383
hsa-miR-891b	-3.185	0.001449	0.031132	0.671202
hsa-miR-572	-3.176	0.001491	0.031771	0.671202

hsa-miR-466*	-3.164	0.001557	0.032892	0.67221
hsa-miR-6814-5p	-3.155	0.001603	0.033592	0.67095
hsa-miR-3612*	-3.152	0.001621	0.033705	0.668934
hsa-miR-20a-3p	-3.149	0.00164	0.033829	0.657848
hsa-miR-377-5p	3.141	0.001686	0.034506	0.670446
hsa-miR-4253*	-3.127	0.001764	0.035804	0.666415
hsa-miR-6827-5p	-3.107	0.001889	0.037573	0.650038
hsa-miR-3143	3.107	0.001893	0.037573	0.670194
hsa-miR-5001-5p*	-3.106	0.001895	0.037573	0.676241
hsa-miR-4722-3p	3.102	0.001925	0.037892	0.666163
hsa-miR-4769-5p	3.090	0.002003	0.03912	0.657596
hsa-miR-6741-5p	3.082	0.002059	0.039863	0.670698
hsa-miR-6796-5p	-3.080	0.002072	0.039863	0.65936
hsa-miR-6807-5p	-3.076	0.002095	0.040014	0.671202
hsa-miR-770-5p	3.073	0.00212	0.040192	0.672965
hsa-miR-190a-3p*	-3.061	0.002203	0.041014	0.675485
hsa-miR-4706	3.061	0.002207	0.041014	0.645251
hsa-miR-6503-5p	3.060	0.002211	0.041014	0.655581
hsa-miR-5093	-3.054	0.002255	0.041516	0.650542
hsa-miR-6790-5p*	-3.051	0.002281	0.041705	0.662383
hsa-miR-7703*	3.042	0.002347	0.042603	0.650542
hsa-miR-130a-3p*	-3.006	0.00265	0.047444	0.64777
hsa-miR-8071	-3.002	0.002681	0.047444	0.679768
hsa-miR-2110	3.001	0.002689	0.047444	0.656085
hsa-miR-4482-3p	3.000	0.002697	0.047444	0.660368
hsa-miR-6751-3p	2.999	0.002705	0.047444	0.650038
hsa-miR-606	-2.997	0.002728	0.04752	0.661628
hsa-miR-423-5p	-2.993	0.002759	0.047741	0.656337

*the markers that were also differentially expressed between PC and non-cancer samples; other markers were only differentially expressed between PC and non-PC samples.

Table S3. Clinical characteristic of the samples used for qRT-PCR.

Variables	qRT-PCR		
	PC (n=17)	ICC (n=8)	N (n=8)
Age	65.0 ± 11.4	63.0 ± 8.2	44.0 ± 9.4
Female	10 (58.8)	3 (37.5)	2 (25.0)
Stage*			
I	2	2	
II	7	1	
III	-	1	
IV	8	4	
CA19-9, U/ml	698±6505.3	93.9±1590.1	6.6±7.44
≤37	4 (23.5)	3(37.5)	8(100)
>37	13 (76.5)	5 (62.5)	-
Overall Survival, months	21.7	15.3	-

* Tumor stages were based on the staging classification of the 7th edition of the American Joint Committee on Cancer.

Variables are expressed as mean±standard deviation, median±standard deviation, or n(%)

PC (pancreatic cancer), ICC (intrahepatic cholangiocarcinoma), N (normal), CA 19-9 (carbohydrate antigen 19-9)
